# Asymmetries and relationships between dynamic loading, muscle strength, and proprioceptive acuity at the knees in symptomatic unilateral hip osteoarthritis

**DOI:** 10.1186/s13075-014-0455-7

**Published:** 2014-12-14

**Authors:** Najia Shakoor, Kharma C Foucher, Markus A Wimmer, Rachel A Mikolaitis-Preuss, Louis F Fogg, Joel A Block

**Affiliations:** Section of Rheumatology, Rush Medical College, Rush University, 1611 West Harrison Street, Suite 510, Chicago, IL 60612 USA; Department of Kinesiology and Nutrition, University of Illinois Chicago, 1200 West Harrison Street, Chicago, IL 60607 USA; Department of Orthopedic Surgery, Rush University, 1653 West Congress Parkway, Chicago, IL 60612 USA; College of Nursing, Rush University, 600 South Paulina Street, Chicago, IL 60612 USA

## Abstract

**Introduction:**

High joint loading, knee muscle weakness, and poor proprioceptive acuity are important factors that have been linked to knee osteoarthritis (OA). We previously reported that those with unilateral hip OA and bilateral asymptomatic knees are more predisposed to develop progressive OA in the contralateral knee relative to the ipsilateral knee. In the present study, we evaluate asymmetries in muscle strength and proprioception between the limbs and also evaluate relationships between these factors and joint loading that may be associated with the asymmetric evolution of OA in this group.

**Methods:**

Sixty-two participants with symptomatic unilateral hip OA and asymptomatic knees were evaluated for muscle strength, joint position sense and dynamic joint loads at the knees. Muscle strength and proprioception were compared between limbs and correlations between these factors and dynamic joint loading were evaluated. Subgroup analyses were also performed in only those participants that fulfilled criteria for severe hip OA.

**Results:**

Quadriceps muscle strength was 15% greater, and in the severe subgroup, proprioceptive acuity was 25% worse at the contralateral compared to ipsilateral knee of participants with unilateral hip OA (*P* <0.05). In addition, at the affected limb, there was an association between decreased proprioceptive acuity and higher knee loading (Spearman’s rho = 0.377, *P* = 0.007) and between decreased proprioceptive acuity and decreased muscle strength (Spearman’s rho = −0.328, *P* = 0.016).

**Conclusions:**

This study demonstrated asymmetries in muscle strength and proprioception between the limbs in a unilateral hip OA population. Early alterations in these factors suggest their possible role in the future development of OA at the contralateral ‘OA-predisposed knee’ in this group. Furthermore, the significant association observed between proprioception, loading, and muscle strength at the affected hip limb suggests that these factors may be interrelated.

## Introduction

Osteoarthritis (OA) of the knee results in vast societal costs and morbidity, and while the epidemiology is well described, the pathophysiology is less well understood. Dynamic joint loads, or loads during physical activity, are central to the biomechanical pathophysiology and progression of knee OA [1-3] and can be assessed using three-dimensional gait analysis. In particular, the peak external knee adduction moment (KAM), assessed through gait analyses has become an accepted surrogate marker of medial knee loading and has been associated with pain [2], severity [1] and progression of medial knee OA. Knee loading during gait is subject to continuous alteration in response to the environment, and a variety of afferent inputs have been demonstrated to be involved. Pain is the most obvious driver of gait alterations, however, other neuromuscular factors have also been shown to be altered in OA, including reduced muscle strength [4,5], proprioception [6-9], and vibratory perception [10,11]. However, the interaction among these factors in the development or progression of OA has not been clearly elucidated.

Muscle strength is hypothesized to provide joint stability and ‘shock absorption’ by both dissipating load and by physiologically distributing it across the joint and it has been observed that persons with OA tend to have weak quadriceps relative to normal controls [12]. As pain leads to disuse as well as muscle inhibition, this weakness has been thought to be a consequence of pain and disuse in OA [13-15]. However, quadriceps muscle weakness has also been observed in subjects who have radiographic evidence of structural knee OA but who are asymptomatic clinically [5], and weakness is also associated with incident symptomatic knee OA [16]. Hence, it is possible that muscle weakness itself contributes to aberrant dynamic joint loading and hence acts as a potential cause as well as the commonly assumed consequence of OA. However, this has not yet been demonstrated systematically and some investigations suggest that increased muscle strength could be detrimental in OA [17]. Understanding the role of muscle strength in OA is important since it is a modifiable factor in the disease.

Proprioception is defined as an awareness of position or detection of movement in space. Proprioceptive deficits have been noted at both the affected and unaffected knees of those with unilateral knee OA [6-9]. Since muscle spindles are proprioceptors, primary muscle pathology could account for these deficits [18-20]. In addition, proprioception, either through its relationship with muscle strength or independently because of the importance of sensory input in preventing sudden and excessive joint impact during walking [21,22], may be related to joint loading; though the role that proprioception plays in aberrant joint loading, if any, has not been confirmed.

We previously observed that patients with end-stage unilateral hip OA who require total hip replacement have a substantially increased risk of developing end-stage OA in their contralateral knee (knee of the opposite limb from hip replacement) relative to their ipsilateral knee [23] and this finding has been confirmed by others [24]. Moreover, we demonstrated that prior to their hip replacement, those with end-stage unilateral hip OA have a higher peak KAM at the contralateral knee compared to the ipsilateral knee that (1) is present in an early phase, when the knees are asymptomatic [25], (2) is higher than those in age-matched controls [26] and (3) is maintained up to two years after successful hip replacement surgery, when subjects’ hips are pain free [26,27]. Thus, these patients develop a gait adaptation wherein their asymptomatic contralateral knees are subjected to long-term overloading and are at risk of progressing to symptomatic OA. Muscle strength and proprioception are neuromuscular factors that may be involved in this gait adaptation. The pattern of OA progression in unilateral hip OA has provided a unique group with which to study factors involved in OA pathogenesis, because one knee is relatively ‘OA-predisposed’ compared to the other knee. Furthermore, if this population is studied early, prior to the onset of significant knee symptoms, then OA-related factors can be assessed without the confounding influence of knee pain that affects studies of patients with symptomatic knee OA.

Thus, aberrant dynamic joint loading [1-3], quadriceps muscle weakness [5,18], and decreased proprioception [6-9] have each been implicated independently in the pathophysiology of knee OA, and appreciating the interactions among these factors may aid in our understanding of the biomechanical pathophysiology of knee OA. Nonetheless, the relationship of each of these factors with the others has not been clearly defined. Moreover, pain, the cardinal clinical manifestation of OA, can modify each of these factors [8,14,15,28,29] and thereby confound evaluation of the role each factor plays in symptomatic OA as well as the interpretation of any interaction among the factors.

The purpose of the current study was to evaluate the contributions of muscle strength and proprioception to the loading asymmetries observed in this model of pre-symptomatic knee OA. The cohort of participants in this study had unilateral hip OA but were essentially asymptomatic at the knees so that the relationships could be evaluated early and with minimal influence from knee pain. Our hypotheses for this study were that asymmetries in periarticular muscle strength and proprioception, specifically diminished muscle strength and proprioceptive acuity in the contralateral limb to the affected hip, are present early in participants known to be at high risk of developing OA. In addition, we hypothesized that muscle strength and proprioception would be directly related to one another and both inversely related to dynamic knee loading.

## Methods

### Subjects

This study was approved through Rush University’s institutional review board for studies involving human subjects, and written informed consent was obtained from all subjects. Detailed enrollment criteria and demographics were previously described for this cohort of subjects [25]. Briefly, inclusion criteria included the presence of unilateral *symptomatic* OA of the hip, which was defined by the American College of Rheumatology’s Clinical Criteria for Classification and Reporting of OA of the hip [30,31] and by the presence of at least 30 mm of pain (on a 100 mm scale) while walking (corresponding to question 1 of the visual analog format of the hip-directed Western Ontario and McMaster Universities Arthritis Index (WOMAC)) [32]. Radiographic OA of the index hip was documented by anterior-posterior radiographs of the pelvis, of grade greater than or equal to 2 as defined by the modified Kellgren-Lawrence (KL) grading scale [33]. During screening, all eligible subjects denied pain at the contralateral hip or either knee. Subjects were further excluded if they demonstrated symptomatic OA of the contralateral hip or of either knee, with presence of pain defined as a response of greater than 30 mm (of 100 mm) while walking (corresponding to question 1 of the visual analog scale format of the site-directed WOMAC), or if there was evidence of *radiographic* OA of the contralateral hip or either knee in excess of grade 3 according to the modified KL scale. The study was designed to enroll an equal number of participants with ‘mild to moderate’ hip OA and ‘severe’ hip OA, defined as a group with KL 4 radiographic changes at the affected hip OA or those pre-hip replacement surgery.

### Radiographs

Radiography of the pelvis and weight-bearing knees were performed in each subject. X-rays were scored for KL grade [34] by experienced readers (NS, JAB).

### Pain assessment

Subjects completed the WOMAC visual analog scale for evaluation of pain at the hips and knees. The WOMAC is the current standard in the analysis of pain and function in lower extremity OA [35]. The total WOMAC pain score is a scale of 0 to 500 mm and was normalized to a 100 mm scale.

### Gait analyses

Bilateral gait assessment was performed using a published marker set and standard methods [36,37]. Passive retroreflective markers were placed on bony landmarks of each lower extremity: the most superior point of the iliac crest, the greater trochanter, the lateral knee joint line, the lateral malleolus, the lateralmost point on the calcaneus, and the head of the fifth metatarsal. A 4-camera optoelectronic camera system (Qualisys, Gothenburg, Sweden) recorded the three-dimensional marker position. Three-dimensional positions of the joint centers were determined based on these marker positions and anthropometric measurements. The magnitude and location of the ground reaction force (GRF) were measured with a multicomponent force plate (Bertec, Columbus, OH, USA). The position and force data were then utilized to calculate three-dimensional external moments using inverse dynamics and processing software developed by CFTC (Computerized Functional Testing Corporation, Chicago, IL, USA). The external moments (Nm) are normalized to the subjects body weight (BW) multiplied by height (Ht) times 100 (%BW*Ht) [38]. Subjects walked with their usual walking shoes at a self-selected normal speed on a force plate located under a 2-inch thick wooden pressboard covered with linoleum. Three trials were performed for each limb. Speed-matched trials at normal walking speed from each limb were chosen for comparison.

The primary gait endpoint was the peak KAM, which reflects the distribution of medial to lateral compartment knee loading during walking. The peak KAM was defined as the external adduction moment of greatest magnitude during the stance phase of the gait cycle.

### Muscle strength analyses

Concentric and eccentric isokinetic strength of knee extensors and flexors were tested using a Biodex™ (Shirley, NY, USA) isokinetic dynamometer. The measurements were performed with the subject in seated position and hips flexed to 90 degrees. Restraints were applied across the waist and an ankle strap proximal to the medial malleolus secured the tibial pad of the force transducer. The axis of rotation of the dynamometer was aligned with the axis of rotation of the knee. Subjects were instructed to fold their arms across their chest. After two test contractions, subjects underwent five maximal concentric and eccentric contractions for knee flexion and extension at a speed of 60 degrees per second with testing range of motion between 0 (full extension) and 80 degrees of flexion. There was a rest period of 10 seconds between successive concentric and eccentric contractions. Peak torque measurements (Nm) were used to represent maximum muscle strength and were divided by BW (kg). The reliability for muscle strength measurements (normal subjects tested on consecutive days) was high with an intraclass correlation coefficient (ICC) of 0.95 to 0.99.

### Proprioception analyses

Proprioception was evaluated as joint position sense at the knee with the passive extension-active replication method using the Biodex™ isokinetic dynamometer (Shirley, NY, USA) [21], which was calibrated using a goniometer for each subject and then provided angular measurements through its software system. Subjects were asked to close their eyes. The subject was in seated position and hips flexed to 90 degrees. Subjects had a handheld device with a push button. The subject’s leg was *passively* extended by the technician, at a rate of approximately 10 degrees per second, to an angle of 45 degrees flexion alternating with an angle of 60 degrees flexion. The angle was maintained for five seconds and then the leg was returned passively to the starting position of flexion. The subject was asked to *actively* extend the leg to reproduce the index angle and press the button on the handheld device when they felt they had reached the appropriate angle. The degrees difference between the starting index angle and the reproduced angle reflected the subject’s ability to estimate angular position accurately (lower number - approximately better proprioceptive acuity). The subjects underwent five repetitions at each angle and the results were evaluated as the mean absolute error of the trials. The reliability for proprioception measurements (normal subjects tested on consecutive days) was fair with an ICC of 0.67 to 0.73.

### Statistical analyses

Statistical analyses were performed using IBM SPSS Statistics 22 (PASW 22, SPSS Inc., IBM, Armonk, NY, USA). Paired samples *t* tests were used to evaluate for asymmetries between the knees in joint loading, muscle strength and proprioception. Since all comparisons were paired and between individual participants’ two knee/limbs, there was not a need to adjust for common confounders that may be necessary when comparing independent groups. Spearman correlations were used to evaluate bivariate correlations between these factors. Since it is possible that asymmetries may develop during later stages of hip OA, subgroup analyses were also performed. The subgroup included those with ‘severe’ hip OA, those with KL grade 4 radiographic disease of the affected hip or those that were pre hip replacement surgery.

#### Power analyses

Little previous data are available regarding differences in muscle strength and proprioception between limbs. Previous studies with loading have suggested a difference ranging from 0.4 to 0.5% body weight*height in the KAM between limbs of pre total hip replacement and post total hip replacement subjects [27]. With 54 subjects, if the true difference in the mean response of matched pairs is 0.35% body weight*height, we will be able to reject the null hypothesis with greater than 80% power, based on a two-sided test with a significance level of 0.05. In terms of correlations, however, we estimate that the minimum meaningful correlation between any two of these parameters would correspond to a correlation coefficient of 0.3. Fifty-four subjects will insure 80% power for detecting a population correlation coefficient of 0.3, based on a two-sided test with a 0.05 significance level.

## Results

One hundred and twenty-four subjects were screened, and 62 subjects fulfilled the inclusion/exclusion criteria and completed the study. Twenty-five of these 62 participants were considered to have ‘severe’ hip OA based on predetermined criteria (*see Methods*). Gait data were available on 58 subjects (some missing due to malfunction of the gait testing system during their visits), muscle strength data on 55 and proprioception data on 54 of the subjects (missing data were secondary to time limitations for participants and inability to stay for complete study visit as well as system malfunction for one participant’s proprioception test). Detailed demographic data have previously been reported on this study group [25]. Subjects had a mean age (± standard deviation (SD)) of 62 ± 11 years. There were 26 males and 36 females. Seventeen participants had KL grade 2 severity at the affected hip, 21 had KL 3, and 24 had KL 4. At the ipsilateral knee, KL distribution was 20 KL 0, 22 KL 1, 18 KL 2, and 2 KL 3. At the contralateral knee KL distribution was 16 KL 0, 21 KL 1, 21 KL 2, and 4 KL 3.

In terms of asymmetries between the knees, results are summarized in Table 1. As we previously reported in this group, peak KAM was approximately 10% higher at the contralateral knee compared to the ipsilateral knee (*P* = 0.029) [25]. In addition, quadriceps muscle strength was greater at the contralateral limb compared to the ipsilateral limb (*P* = 0.006). No significant differences were observed in proprioceptive acuity between the contralateral and ipsilateral knees (*P* = 0.486).

**Table 1:** **Paired comparisons of muscle strength, proprioception and knee loading between the limbs**

	**Ipsilateral limb**	**Contralateral limb**	***P*** **value**
**Unilateral hip OA (entire group, n = 62)**			
Quadriceps muscle strength (Nm/kg)	0.95 ± 0.35	1.08 ± 0.41	0.006^*^
Proprioception (degrees error)	4.7 ± 2.8	5.0 ± 3.0	0.486
Dynamic loading/KAM (%BW*Ht)	2.23 ± 0.81	2.46 ± 0.71	0.029^*^
**End-stage hip OA (subgroup, n = 25)**			
Quadriceps muscle strength (Nm/kg)	0.89 ± 0.23	1.08 ± 0.39	0.010^*^
Proprioception (degrees error)	4.8 ± 3.1	6.0 ± 3.3	0.022^*^
Dynamic loading/KAM (%BW*ht)	2.14 ± 0.80	2.53 ± 0.85	0.022^*^

All values are mean ± standard deviation (SD); ^*^ *P* <0.05. OA, osteoarthritis; Nm, external moments; KAM, knee adduction moment; BW, body weight; Ht, height.

In subgroup analyses in those with severe hip OA only (n = 25), the loading and muscle strength asymmetries remained (Table 1), but in addition, proprioceptive acuity was more than 20% worse in the contralateral knee compared to the ipsilateral knee, 6.01 ± 3.3 vs. 4.8 ± 3.1 degrees error, respectively (*P* = 0.022).

**Figure 1:**
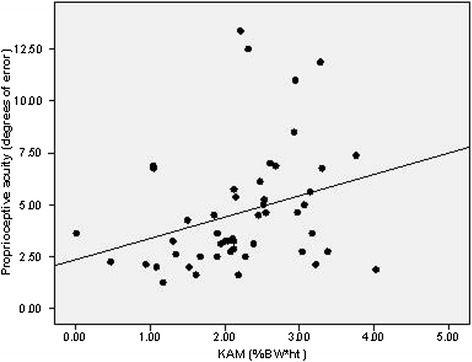
**The association between proprioception (joint position sense) and knee joint loading (KAM) at the ipsilateral limb of participants with unilateral hip osteoarthritis (OA) (Spearman’s rho = 0.377,** ***P*** **= 0.007).**

**Figure 2:**
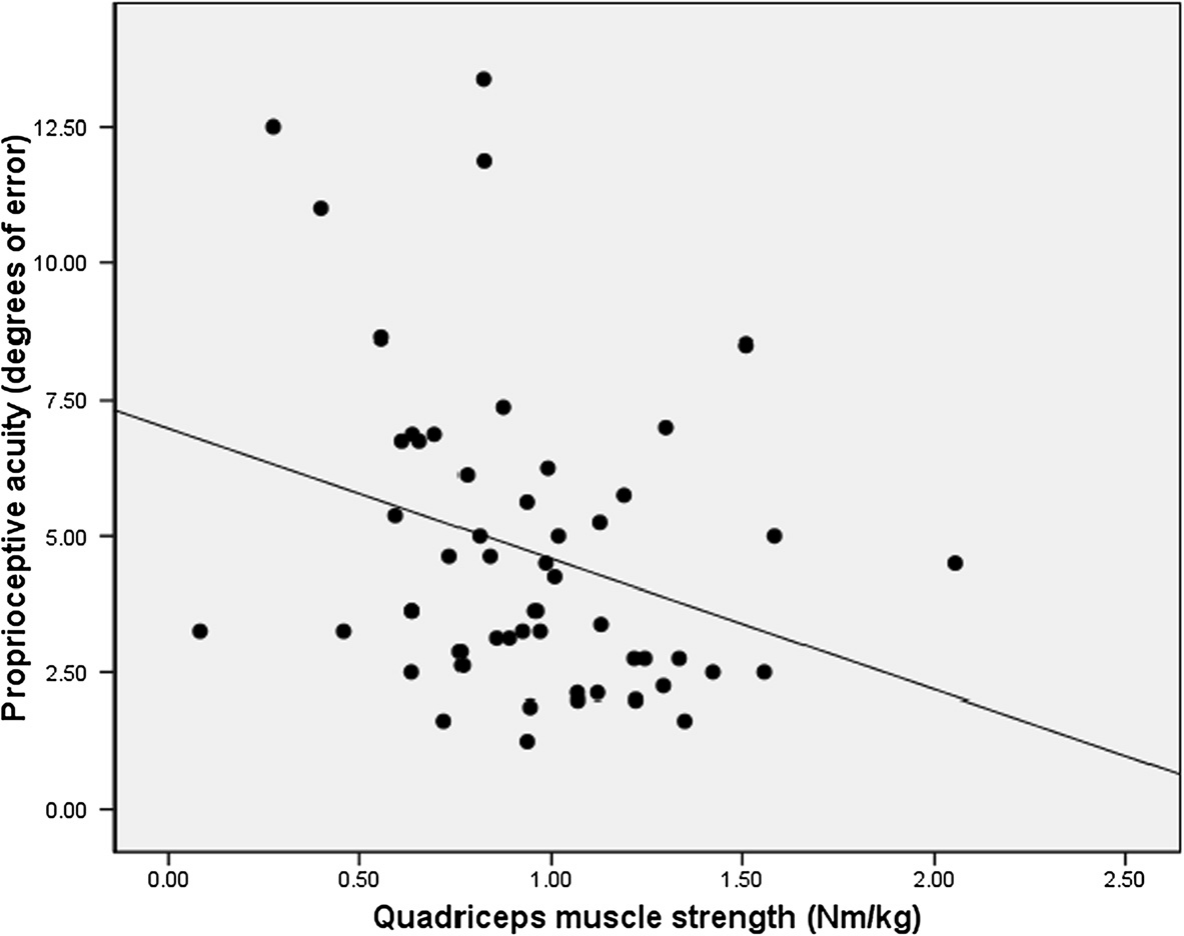
**The association between proprioception (joint position sense) and quadriceps muscle strength at the ipsilateral limb of participants with unilateral hip osteoarthritis (OA) (Spearman’s rho = -0.325,** ***P*** **= 0.017).**

For evaluation of relationships between loading, muscle strength and proprioception, the limbs were evaluated separately. At the limb with the affected hip, there was an association observed between the peak KAM and proprioception (Spearman’s rho = 0.377, *P* = 0.007; Figure 1), thus, decreased proprioceptive acuity was associated with higher knee loading. At the affected hip limb, there was also an inverse association observed between proprioception and muscle strength (Spearman’s rho = −0.328, *P* = 0.016; Figure 2), that is, decreased proprioceptive acuity was associated with weaker quadriceps muscle strength. At the affected hip limb, there was no relationship observed between maximal muscle strength and loading (Spearman’s rho = 0.018, *P* = 0.898). Conversely, there were no associations present between these factors in the limb of the unaffected hip: proprioception with loading (Spearman’s rho = − 0.160, *P* = 0.463), proprioception with muscle strength (Spearman’s rho = −0.214, *P* = 0.120), and muscle strength and loading (Spearman’s rho = −0.154, *P* = 0.281).

Of note, there were no significant associations observed between WOMAC pain at the affected hip and either ipsilateral or contralateral knee loads, quadriceps strength, or proprioceptive acuity (*P* >0.05 for all correlations).

## Discussion

Those with unilateral hip OA have been shown to have relatively higher risk of contralateral knee OA compared to ipsilateral knee OA [23,24]. This study demonstrates significant lower-limb neuromuscular asymmetries among patients who have unilateral hip OA, but who are asymptomatic or have minimal symptoms at the knees. The contralateral knee in this group has substantially stronger quadriceps, and, in those with advanced structural hip OA, worse proprioceptive acuity than the ipsilateral knee. Significant relationships between proprioception and knee loading as well as between proprioception and muscle strength were demonstrated in this study as well.

We have previously demonstrated that persons with unilateral hip OA have increased dynamic loads at the contralateral knee compared to the ipsilateral limb knee [25,27]. We have shown that these asymmetries are present early [25] and persist up to two years after successful hip replacement surgery has rendered the hip pain free [27]. We felt that the initial asymmetry in loading could be a response to pain, an attempt to unload the affected hip limb. We hypothesized that the persistent asymmetry in a pain-free state might be related to a gait adaptation resulting from alterations in neuromuscular factors such as muscle strength and proprioception, which this study suggests may be the case.

Here, the identified asymmetries in quadriceps strength are unexpected, with greater strength at the contralateral knee compared to the ipsilateral knee. It is possible that the observed relative weakness of the ipsilateral limb may have been related to chronic pain and disuse or acute pain of the affected limb during testing; although subjects with knee pain were excluded, hip pain might have referred distal effects. In addition, although WOMAC pain at the affected hip did not correlate with factor deficits, pain during testing may; no such pain was observed, but a limitation of this study is that we did quantitatively evaluate pain during testing. It is also possible that the contralateral limb had been relatively strengthened in an attempt to compensate for mechanical inadequacy of the ipsilateral limb secondary to painful hip OA. Furthermore, although most studies consider muscle weakness to be a risk factor for OA, one study did suggest that greater muscle strength was associated with OA progression in malaligned knees [17]. Thus, if malalignments were present, based on that study, the greater strength at the contralateral limb could have predisposed participants to progressive OA at the contralateral knee. A limitation of the current study is that we do not have information on alignment.

Our first hypothesis was partially supported in that proprioceptive acuity was found to be diminished in the contralateral knee compared to the ipsilateral knee of the ‘severe’ unilateral hip OA group. It should be noted that the absolute difference between the knees was small, and therefore, the clinical significance of this difference is unclear. However, OA is a process that progresses slowly over decades, and small differences that may be inconsequential over short periods of time may have accentuated impact when assessed over years of chronic use. This is of interest because proprioceptive deficits have been associated with presence of knee OA [7,8,39] and have shown possible associations with physical function [40], functional decline and pain in OA [41,42]. In this study of ‘early OA’, the contralateral (‘OA-predisposed’) knee already had proprioceptive deficits relative to the ipsilateral knee, providing evidence that proprioceptive deficits may *precede* the onset of symptomatic knee OA. The fact that this asymmetry was only noted in those in the ‘severe hip OA’ group, may be because these asymmetries are related to the severity and duration of the unilateral hip OA.

A direct relationship between strength and loading was not observed in this study, and therefore, our second hypothesis was not supported. Previous investigators have similarly been unable to demonstrate a relationship between quadriceps strength and the peak KAM in OA [43]. It may be that maximum quadriceps muscle strength is not a significant contributor to the KAM; other muscle groups such as hip adductors may have a larger role [44,45], although recent studies have been inconsistent regarding whether hip muscle strengthening reduces the peak KAM [44,46]. Or it may be that muscle strength only affects loading in extreme degrees of muscle weakness, which were not present in this study population. Alternatively, muscle strength may operate through a mechanism that is not reflected in KAM. For example, it is possible that greater strength in the presence of poorer somatosensory afferent perception (such as proprioception) might predispose the joint to greater long-term damage than joints compressed by weaker muscles; however, since some studies do suggest the protective role of muscle strength in OA [47,48], these mechanistic relationships require further study.

A novel finding of this study is that decreased proprioceptive acuity was found to be significantly associated with decreased muscle strength in the affected hip limb. This relationship has not been directly demonstrated in the literature, although others have suggested that perhaps a muscle strength-proprioception interaction is important in predicting functional decline in OA, with muscle weakness having a greater impact in those with poor proprioceptive acuity [40]. Thus, although our observation was in the affected hip alone, perhaps there may be some connection between proprioception and muscle strength that became apparent in the presence of hip pathology at the affected limb but is not yet apparent at the contralateral, unaffected limb, in our model. The physiologic basis for the observed association needs to be better understood and the interactions between these factors need to be further elucidated.

This study also supported the initial hypothesis regarding a significant inverse relationship between proprioceptive acuity and knee loading. Options are that high loading affects joint mechanoreceptors and thereby, proprioception; conversely, poor proprioception could lead to impaired sensory control of the limb during walking, and therefore, increased dynamic joint loads. We previously showed a similar relationship between vibratory perception, another somatosensory measure, and dynamic joint loading [11]. To evaluate this further, interventional studies targeting proprioception may be able to evaluate the resultant effects on joint loads.

This model exploits the use of bilateral limbs in the same individuals and all comparisons are relative. ‘OA-predisposed’ knee does not necessarily refer to predisposed compared to all persons, but relative to the affected limb knee. Further, end-stage knee OA in the contralateral limb is not inevitable among these patients, only that if OA were to develop, it would more likely occur at this contralateral limb relative to the ipsilateral limb.

It is important to note that significant relationships between proprioception, muscle strength, and loading were only observed in the limb with the affected hip. The reasons for the lack of concordance between the limbs are unclear.

We used a clinically relevant definition of knee OA in this study. Several of the participants did have radiographic evidence of structural degeneration in their knees, but based on standard definitions, would be considered ‘asymptomatic’. This is a practical approach since there is a well-known discordance between structural degeneration (so-called *radiographic* OA) and clinically significant OA, and it is likely that many persons in this age group would have incidental radiographic findings of OA.

## Conclusions

This study provided a unique assessment of the role of neuromuscular factors in OA pathogenesis. In unilateral hip OA, this study demonstrates increased muscle strength and greater proprioceptive deficits at the contralateral, ‘OA-predisposed’ knee, suggesting that these OA-related factors are aberrant in the absence of significantly symptomatic disease at the knees. Thus, these risk factors for knee OA are not entirely a consequence of knee pain; it may be prudent to address asymmetries in these factors early in the disease process. Furthermore, this study demonstrated associations between knee proprioception and both knee muscle strength and loading. Since these associations were present at the affected hip limb only, future studies should evaluate potential factors that may contribute to the manifestation of these relationships.

## Abbreviations

BW: body weight
GRF: ground reaction force
Ht: height
ICC: intraclass correlation coefficient
KAM: knee adduction moment
KL: Kellgren-Lawrence
Nm: external moments
OA: osteoarthritis
WOMAC: Western Ontario and McMaster Universities Arthritis Index
